# Why We Need Greater, Not Less, Access to Reduced-Risk Nicotine Products

**DOI:** 10.7759/cureus.90452

**Published:** 2025-08-19

**Authors:** Ian M Fearon, Marina A Murphy

**Affiliations:** 1 Scientific Research, whatIF? Consulting Ltd, Harwell, GBR; 2 Scientific Affairs, HAYPP Limited, London, GBR

**Keywords:** accessibility, cigarette smoking, electronic cigarettes (e-cigarettes), reduced risk nicotine products, tobacco harm reduction

## Abstract

Cigarette smoking is the leading preventable cause of death and disease. Prolonged and repeated inhalational exposure to the chemical toxicants found in cigarette smoke is a known cause of cardiovascular disease, chronic lung disease, and cancer. Novel nicotine products, including e‑cigarettes and tobacco‑free nicotine pouches, reduce exposure to toxicants among those who smoke and switch to their use, reducing the risk of smoking‑related disease. Implementation of national strategies to reduce smoking‑related death and disease increasingly acknowledges the support that novel nicotine products can give to smoking prevention efforts. However, for such strategies to be successful, and for countries to achieve their ‘SmokeFree’ ambitions, these products need to be widely accessible to consumers. In a recent paper, Aremu and Ogugua called for restrictions on access to electronic cigarettes (e‑cigarettes) or electronic nicotine delivery systems (ENDS). While prescription‑only regulatory models were advocated, these risk constraining accessibility to those who smoke and have been shown to cause increases in criminal activity, systemic violence, and health risks associated with the proliferation of unregulated nicotine products. Prescription‑only regulation should be avoided due to the potential for these significant unintended consequences, although there is a place for prescription products in co‑existence with products available through general consumer routes. Instead of banning or placing restrictions on purchasing, the use of reduced-risk nicotine products among unintended users, such as youth, can be mitigated through innovation and technology. This would ensure that access to reduced-risk products among those who smoke would best serve tobacco harm reduction efforts and population health.

## Editorial

Introduction

Cigarette smoking is the world’s leading preventable cause of death and disease. Long‑term, repeated exposure to the thousands of chemicals in cigarette smoke claims over 7 million lives every year [1], and smoking has accounted for more than 175 million deaths across the world over the past three decades [2]. Globally, over 1 billion people smoke combusted tobacco products, and novel approaches are, therefore, needed to reduce smoking‑induced incidences of cardiovascular disease, chronic lung disease and cancer. In a recent paper, Aremu and Ogugua called for restrictions on access to electronic cigarettes (e‑cigarettes) or electronic nicotine delivery systems (ENDS). This proposal suggests reclassifying e‑cigarettes as prescription‑only products [3]. Here, we propose that such restrictions are counterintuitive to reducing the morbidity and mortality associated with cigarette smoking, and that what those who smoke need is greater, not less, access to products that can support public health goals and ‘SmokeFree’ ambitions.

Why e-cigarettes and other reduced-risk nicotine products are a solution, not the problem

The use of e‑cigarettes, or other reduced‑risk nicotine products, such as tobacco‑free nicotine pouches (TFNPs), poses less of a risk to human health than cigarette smoking [4-7]. These products either heat a nicotine‑containing liquid or a tobacco substrate in a consumable, or, in the case of TFNPs, do not involve heating nicotine or tobacco at all. Therefore, consumers are exposed to significantly lower levels of the toxicants responsible for smoking‑related disease [4-6]. When placed on a ‘continuum of risk’, novel nicotine products lie far away from tobacco cigarettes [7-10]. Many substantive reviews show that the use of these novel products is significantly less harmful than cigarette smoking [4-6], including a recent systematic review which found that the disease risk associated with TFNPs is even lower than that of nicotine replacement therapy (NRT) [9]. A number of global public health bodies now support a tobacco harm reduction (THR) approach to reducing the morbidity and mortality associated with combusted tobacco use and improving public health. These include the United States Food and Drug Administration [11], the United Kingdom (UK) Office for Health Improvement Disparities (OHID), formerly known as Public Health England and the Department of Health [12], the Royal College of Physicians [13], Health Canada [14], and the New Zealand Ministry of Health [15]. The fundamental aim of THR is to support individuals who are unwilling or unable to stop smoking to switch to reduced‑risk nicotine products, exclusive use of which reduces their exposure to harmful chemical toxicants [13,16,17]. Promoting complete switching to the use of reduced-risk products will lead to improvements in both individual and population‑level health. Regulatory approaches which are more permissive to THR may have downsides, including youth uptake, dual use and prolonged smoking, or shifting dependence to other tobacco/nicotine products. However, modelling studies have shown that the availability of e-cigarettes represents a significant population‑level benefit [18,19] and may complement other tobacco control interventions [20]. In contrast, e‑cigarette restrictions may drive cigarette use and illicit markets [21].

Accessibility is a key factor in THR. Access to reduced-risk products among those who smoke is thwarted by product bans, and this has a profound impact on THR efforts. Many countries now embrace a THR approach in their ‘SmokeFree’ ambitions [14,15,22,23]. Those countries with high adoption rates of alternative nicotine products have lower smoking rates than those that do not [24]. Sweden stands out as an exemplar of THR in practice. Here, the use of smokeless tobacco and nicotine products has driven down smoking prevalence to the lowest level globally, and Sweden has the lowest rates of smoking‑related disease in Europe [25]. Other countries are being urged to adopt THR policies to reduce the harms associated with tobacco smoke in their populations [20,26,27].

E-cigarettes and smoking cessation

Increasing evidence suggests that e‑cigarettes support smoking cessation. The most recent independent, systematic Cochrane review found that there was high‑certainty evidence that nicotine e‑cigarettes increase quit rates compared to NRT [28]. Importantly, e‑cigarettes support smoking cessation among the socially disadvantaged [29], an important factor when considering the sales restrictions proposed by Aremu and Ogugua [3]. Smoking is, after all, most prevalent in socially disadvantaged groups and/or those with comorbidities such as mental illness [29,30]. The proposal to reduce or eliminate online availability of reduced‑harm products makes no allowance for individuals who smoke and are unable to access physical retail locations because they are disadvantaged, have no access to transportation, have a disability, or live in rural areas. It is important to note in this regard that in the US, for example, the prevalence of cigarette smoking is 15.4% among those who live in rural communities, whereas it is only 10.1% among adults who live in urban areas [31]. A reduced access model also does not consider the potential impacts on those on low incomes or without healthcare insurance for whom licensed NRT products are inaccessible. Smoking prevalence among uninsured individuals in the US is 16.8%, close to double the prevalence among adults with private insurance [31]. Restricting access to reduced-risk nicotine products could, therefore, severely hamper efforts to reduce smoking prevalence amongst those sections of society in which smoking prevalence is highest and which arguably need greater, and not less, support and access to reduced-risk nicotine products. It is also noteworthy that the US FDA, which has a stringent nicotine product authorisation process, recently authorised the sale of certain e‑cigarette and TFNP products. These authorisations are based on the FDA’s view that the overall population‑level health benefit of such an authorisation would be net positive, considering, as it does, the competing interests of giving those who smoke access to reduced-risk products and preventing youth access.

How can we tackle youth access to nicotine products without unintended consequences among those who smoke or have quit?

Youth access to nicotine products should be prevented. In the US, where e‑cigarettes are widely accessible both online and in physical retail, youth use is declining and, in 2024, reached its lowest level in almost a decade [32]. Youth smoking rates are also at a historic low [33]. While any youth use of nicotine is a serious concern that should be eradicated, it is important to recognise that smoking‑related death and disease remain the far greater public health challenge. In 2022, the most recent year for which smoking prevalence data are available, an estimated 28.8 million US adults were smoking cigarettes, leading to 480,000 annualised deaths and hundreds of billions of dollars in healthcare spending and lost productivity. In some segments of the US population, such as those aged 65+ or those of low socioeconomic status, smoking declines have either stalled or are reversing. Clearly, more needs to be done, especially if we are to avert the forecasted loss of over 2 billion years of life due to cigarette smoking between 2022 and 2050 [2]. Elimination of cigarette smoking, even by 2050, could result in avoiding 876 million cumulative life years lost and produce gains in life expectancy at birth at a rate not possible by many other public health interventions [2]. Reducing access to products like e‑cigarettes, which are reduced-risk and have a proven ability to help those who smoke switch to a less harmful alternative, is not the solution and could, in fact, exacerbate the problem. As regards youth access to e‑cigarettes and other nicotine products, this can be mitigated by the use of modern age‑verification technologies, which can easily be applied to both online and physical sales. Furthermore, it is not difficult to implement a two‑step process to age‑verify for online sales at both the point of purchase and the point of delivery. Additionally, novel technologies have been developed that embed near‑field communication (NFC) chips into e‑cigarettes, rendering them unusable unless unlocked at the point of purchase and following age verification [34]. An example provided by the e‑cigarette manufacturer JUUL in its 2023 Pre‑Market Tobacco Product Application (PMTA) to the US FDA showed that social sourcing of e‑cigarettes by youth can also be prevented through technological innovation [35].

Is a prescription-only model a workable solution?

A prescription-only model could be beneficial if trained pharmacists could support those who smoke and want to quit smoking using e-cigarettes. However, support from former smokers who quit successfully using consumer products has significant potential in encouraging switching [36]. Furthermore, the implementation of a prescription-only model has the potential for unintended consequences. A real‑world example of what happens when a prescription‑only model for e‑cigarettes is implemented comes from Australia, a country where such a policy was implemented in 2011 and recently revised [37,38]. This policy has fuelled a thriving illicit market for both youth and adults and caused increases in criminal activity, systemic violence and health risks associated with the proliferation of unregulated nicotine products [39,40]. The Australian prescription‑only model has also reduced access to e‑cigarettes among those who smoke and do not wish to access products through illicit channels. This has coincided with a stalling of smoking prevalence in Australia, with the most recent data showing only 0.3% year‑on‑year declines in smoking prevalence [41]. This suggests that a prescription‑only model for e‑cigarettes is not only unworkable but can lead to major unintended consequences. That is not to say that there isn’t a place for medicinally licensed e‑cigarettes that can be accessed by prescription or through pharmacies. Such a product, licensed, for example, via the marketing authorisation application process set out by the UK Medicines and Healthcare Products Agency, in co‑existence with consumer products, could be highly advantageous. It would mean that reduced‑risk products that support smoking cessation are available to consumers who choose to quit smoking without professional support, while arming medical professionals with additional tools to assist those seeking cessation support via more traditional means. Given that e‑cigarettes are proven to be more effective than conventional NRT products in supporting quitting, such a co‑existence could accelerate reductions in smoking prevalence and improve public health.

Conclusions

Overall, e‑cigarettes and other reduced-risk nicotine products can support smoking cessation and are better alternatives to combustible cigarettes. Their widespread availability is helping many countries meet their ‘SmokeFree’ ambitions. If we are to continue to make strides in reducing smoking prevalence and improving population‑level health, we need greater access to reduced-risk nicotine products, not less. We must not lose sight of the overall goal, which is to support THR efforts to end the preventable death and disease associated with combusted tobacco use. We must use whatever technological means are available to prevent youth access without hindering access to adults who smoke. Prescription e‑cigarettes can play a role in achieving ‘SmokeFree’ goals, but would do better in co‑existence with consumer products. The prescription‑only model has been tried and has failed. Policymakers need only look to Australia to understand the severe negative impact such prohibitive e‑cigarette regulation can have. They should instead consider the population health gains observed in countries with THR-permissive regulatory frameworks.
